# Serum levels of RANKL and OPG, and the RANKL/OPG ratio in bisphosphonate-related osteonecrosis of the jaw: Are they useful biomarkers for the advanced stages of osteonecrosis? 

**DOI:** 10.4317/medoral.22128

**Published:** 2017-08-16

**Authors:** Leticia Bagan, Yolanda Jiménez, Manuel Leopoldo, Andrea Rubert, Jose Bagan

**Affiliations:** 1 Associate Professor of Oral Medicine. Universidad Europea de Valencia; 2 Professor of oral manifestations of systemic diseases. Valencia University; 3 Oral and Maxillofacial Surgeon. Consultant. University General Hospital; 4 Associate Professor of Clinical Dentistry. Universidad Europea de Valencia; 5 Professor of Oral Medicine, Valencia University. Head Service of Stomatology and Maxillofacial Surgery, University General Hospital. Fundación para la investigación del Hospital General Universitario de Valencia, Spain

## Abstract

**Background:**

We determined whether serum levels of Receptor Activator for Nuclear Factor κ B Ligand (RANKL), Osteoprotegerin (OPG), and the RANKL/OPG ratio could be useful biomarkers for the severity of oral lesions in bisphosphonate-related osteonecrosis of the jaw (BRONJ).

**Material and Methods:**

A case-control study in which Group 1 consisted of 41 patients with BRONJ due to intravenous bisphosphonates, and Group 2 consisted of 44 healthy control cases. The plasma levels of RANKL and OPG were analyzed by an ELISA assay. The OPG/RANKL ratio was also calculated. We determined if the mean serum values differed among the different stages of BRONJ.

**Results:**

Serum levels of RANKL were lower in Group 1 than in Group 2 (*p* =0.01), and serum levels of OPG were higher in patients with BRONJ than in the controls (*p* =0.006). The ratio of RANKL/OPG was greater in the controls than in Group 1 (*P* >0.01). There were no significant differences in the serum levels of RANKL and OPG among the different stages of osteonecrosis (*P* > 0.05).

**Conclusions:**

Serum levels of RANKL and OPG, and the RANKL/OPG ratio were not valuable biomarkers for determining the severity of oral lesions in patients with BRONJ.

** Key words:**Bisphosphonates, RANKL, OPG, Osteonecrosis.

## Introduction

Medication osteonecrosis of the jaw (MRONJ) is a severe complication that occurs after anti-resorptive treatments for metastatic cancer, multiple myeloma, and osteoporosis. Several drugs have been implicated in this disorder since 2003 when the first series of cases were described (1), the most common of which is bisphosponate (BP) (2-5).

More recently, other drugs such as denosumab (6-8) and anti-angiogenic drugs (9) have been implicated in MRONJ, the latter of which is used for cancer treatment.

The exact mechanism of MRONJ remains unclear; however, many investigations have described and proposed an osteoclast impairment mechanism with a lack of normal resorption capacity. Other potential etiological factors include the role of local infections in the dental and periodontal areas; dental surgery is considered to be the primary precipitating factor for this oral complication related to anti-resorptive drugs (10).

For bisphosphonate-related osteonecrosis of the jaw (BRONJ), the number of doses and accumulation of BPs are important factors underlying the pathogenesis of this disease (10). BPs inhibit the differentiation of osteoclasts (11) and zoledronic suppresses the TNF-α- and RANKL-induced migration of precursors by inhibiting the mevalonic acid pathway (12).

It has been reported that BP decreases Receptor Activator for Nuclear Factor κ B Ligand (RANKL) and increases Osteoprotegerin (OPG) in animal models (13). These phenomena also contribute to the onset and persistence of BRONJ.

Previous studies have attempted to identify a biomarker that could be useful for the management and prognosis of BRONJ (14).

To the best of our knowledge, no clinical studies have compared the stage classification of BRONJ patients with their serum levels of RANKL and OPG. The aim of this study was to analyze differences in serum levels of RANKL and OPG in the different stages of BRONJ as a potential biomarker for the severity of MRONJ.

## Material and Methods

This case-control study was approved by the Ethical Committee of Valencia University (No. H1417371704425). The study was performed in the service of Stomatology and Maxilofacial Surgery, Valencia, and at the University of Valencia, Spain. The patients were divided two groups: Group 1 consisted of 41 patients with BRONJ due to BPs, and Group 2 included 44 healthy control cases. In Group 1, only patients presenting with at least one area of osteonecrosis within stages 1 to 3 were included. In addition, only cases where there was an exposed bone necrotic area or fistula in the bone with or without symptoms and infection were included, so stage 0 patients were excluded. The diagnosis of osteonecrosis was performed following the Ruggiero criteria (10).

All of the patients agreed to participate in the study.

Of the 41 cases, 32 (78.05%) received intravenous zoledronic acid (Zometa® Novartis Pharma SpA, Basel, Switzerland) every 4 weeks for the treatment of metastatic cancer, and 9 (21.95%) were treated with oral BPs for osteoporosis. Group 2 did not have any systemic disease or oral lesions, and did not receive any treatment that could interfere with bone metabolism. In each case, a blood sample was obtained from a peripheral arm vein before starting any treatment after a diagnosis was made. Blood was immediately centrifuged at 3000 rpm for 10 min, and the samples were filtered and stored at -80°C until final analysis. Plasma levels of RANKL and OPG were analyzed using an ELISA assay.

The RANKL and OPG concentrations in the samples were assayed using a commercial enzyme-linked immunosorbent assay (ELISA; Quantikine RANKL and OPG, R&D Systems, Minneapolis, MN, USA). Briefly, plasma samples were loaded onto microplates pre-coated with specific monoclonal RANKL and OPG antibodies. Standards and samples were bound by the immobilized antibody. After washing to remove unbound material, an enzyme-linked polyclonal antibody specific for RANKL and OPG was added to the wells. After a second wash with buffer, the substrate solution was added and incubated for 30 min while being protected from light. The reactions were stopped with the appropriate substrate. The optical density of the color in each well was measured at 450 nm. RANKL and OPG levels were reported as means ± SDs, expressed in pg/mL. The OPG/RANKL ratio was also calculated. For statistical analysis, we used the x2 test to analyze the association between qualitative variables. To determine whether there were differences in the mean of the two different groups, we used the Mann–Whitney test if the values did not follow a normal distribution. If there were more than two groups, we applied the Kruskal–Willis test for non-parametric values. *P* values less than 0.05 were considered statistically significant.

## Results

The clinical data of our patients are summarized in  Table 1. Regarding the plasma RANKL levels, the highest values were found in the controls, whereas the lowest were detected in Group 1, representing patients with osteonecrosis (*p* = 0.01). When considering plasma levels of OPG, the highest levels were found in the group with osteonecrosis (Group 1) (*p* = 0.006). The ratio of RANKL/OPG was greater in the controls than in Group 1 (*p* < 0.01) ( Table 2). Based on the comparison of serum levels of RANKL and OPG, and the RANKL/OPG ratio among the different stages of osteonecrosis, no significant differences were observed (*p* > 0.05). We also did not find differences in OPG and RANKL/OPG based on the comparison of the BP administration route (intravenous or oral) or location of the lesion on the mandible or upper jaw (*p* > 0.05) ( Table 3).

**Table 1:**
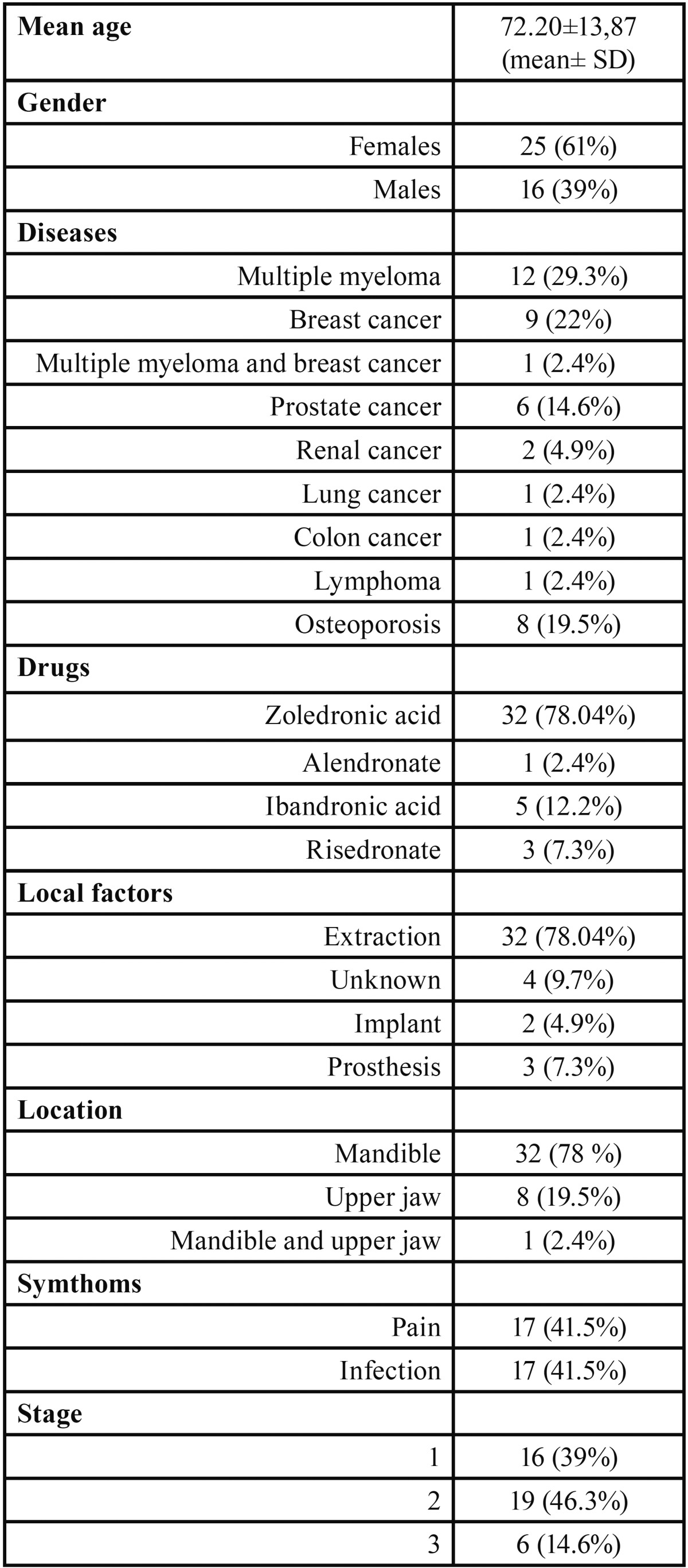
Description of clinical data in Group 1.

**Table 2:**
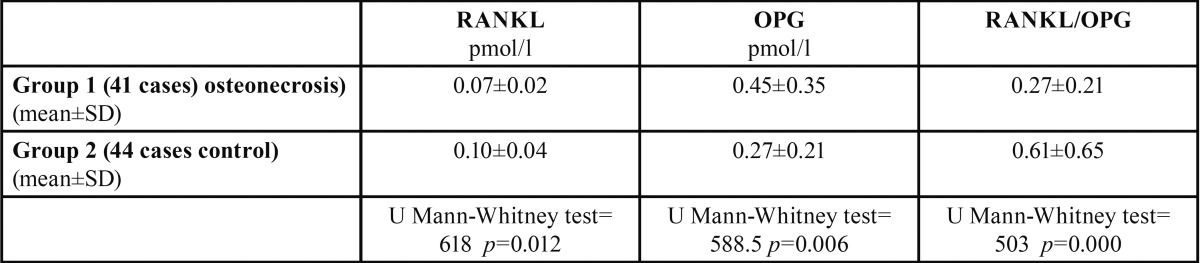
Comparison of serum RANKL, OPG, and the RANKL/OPG ratio between groups.

**Table 3:**
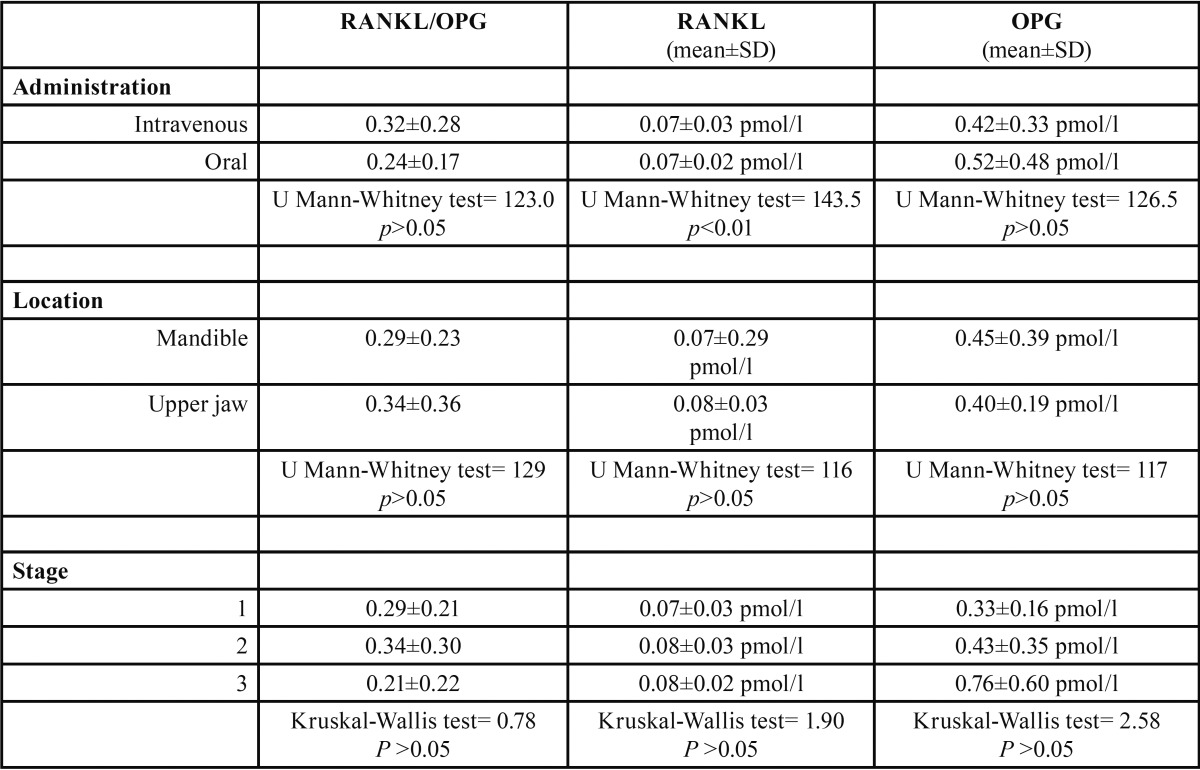
Differences in serum RANKL, OPG, and the RANKL/OPG ratio depending upon the type of administration, location, and stage of osteonecrosis in a group of 41 cases with BRONJ.

## Discussion

BPs have been used for many years (15), Zoledronic acid is a potent antiresorptive BP used to manage osteolysis or hypercalcemia due to malignant diseases such as metastatic cancer and multiple myeloma. BPs are also commonly used for the treatment of metabolic bone diseases such as osteoporosis, in which there are bone resorption processes (16). In addition to diminishing the bone resorption process, BPs promote the apoptosis of osteoclasts (17).

In summary, BPs can inhibit the progression of bone metastasis as well as other skeletal tumor burdens through their ability to inhibit osteoclast function (18).

When BPs are within the bone, ion exchange and a phenomenon of chemisorption occurs between the phosphonate groups of BPs and the inorganic phase of the extracellular matrix, as described by Sandhöfer *et al.* (19).

Non-amino-BPs are metabolized by osteoclasts, generating an adenosine triphosphate (ATP) analogue that leads to decreased functional ATPs, which promotes osteoclast apoptosis (20).

For amino-BPs, osteoclast apoptosis is caused by inhibition of the mevalonate pathway and protein prenylation. Inhibition of farnesyl pyrophosphate synthase results in the suppression of geranylgeranylation and farensylation of small G-proteins. As a consequence, the inhibition of Rab, Ras, and RhoA has a strong effect on cancer patients through various mechanisms such as inducing apoptosis, cell cycle arrest, anti-migration, anti-invasive, and antiangiogenic effects (21).

More recently, other drugs that inhibit the bone resorption process have been prescribed to these patients such as denosumab, which is a selective antagonist of RANKL (22) that also causes osteonecrosis of the jaw, not only when used for oncology but also for the treatment of osteoporosis (6,23).

Its mechanism of action differs from the primary mechanism of BP. There is a continuous attempt to identify biomarkers that can be used to predict the evolution of MRONJ or to identify more severe clinical cases of osteonecrosis.

McGowan *et al.* (14) summarized the possible biomarkers of osteonecrosis in the jaw. Current biomarkers include nadir WBC <1000/mL, the Treg/Th17 ratio, GCF IL-1B, serum antibody levels against Porphyromonas gingivalis, and serum levels of VEGF, ESR, CRP, and CTX. (14).

However, Ruggiero *et al.* (10) stated that the use of systemic markers has not been validated. Many studies have explored various biomarkers, but without clinical utility.

For the C-terminal cross-linking telopeptide of type I collagen, previous reports (10,24,25) have noted that serum CTX is a biological marker of ONJ, whereas other studies have not reported this finding (26).

Friedlander *et al.* (27) concluded that only a limited number of studies have evaluated the prevalence of depressed CTX levels of less than 150 pg/mL among OBP recipients and that only a very small minority of such patients subsequently developed MRONJ after dental extraction. We do, however, believe that the CTX test is potentially a valuable aid in the clinical decision-making process, but not an absolute determination of an individual’s risk of developing MRONJ.

We found that plasma and saliva IL-6 values were higher in patients with BRONJ than in the controls, suggesting that IL-6 may be a useful tool for monitoring the severity of BRONJ (28).

Another common finding in osteonecrosis is bacterial infection of the exposed bone, mainly with the presence of Actinomyces (29).

Anav- Lev *et al.* (30) confirmed that Actinomyces infection is a etiological factor in BRONJ. We found that plasma and saliva oxidative stress levels were higher in patients with BRONJ than in controls, and may be useful for monitoring. Reactive oxygen species are common in inflammatory processes, and the oxygen radical products are important for killing microorganisms (31).

Circulating sRANKL levels may also be modulated by BPs (32). However, previous studies that analyzed changes in RANKL after BP treatment were performed in animals.

Çankaya *et al.* (13) treated a group of rats with zoledronate and compared the results with two control groups: one injected with saline solution and another with no injection of any substance or drug. The authors found that RANKL values in the mandible were decreased in the zoledronic group. By contrast, they observed increased OPG levels in the zoledronic group. Another previous report described changes in RANKL/OPG gene expression after BP treatment.

Koch *et al.* (33) observed enhanced RANKL/OPG gene expression after stimulation by BPs in an in vitro study.

Di Nisio *et al.* (34) proposed that bacteria in the necrotic area could trigger the RANK/RANKL/OPG signaling pathway. We explored whether serum levels of RANKL and OPG, or the RANKL/OPG ratio had any relationship with the different stages of MRONJ. This observation has never been described in patients with osteonecrosis.

We only selected cases with necrotic bone exposure to account for any possible confusion with stage 0 diagnoses.

Some authors (35) have commented on the non-specific findings of stage 0, which could complicate the final stage classification of MRONJ.

Mawardi and Woo (36) suggested that it may be useful to remove Stage 0 entirely and only maintain stages 1, 2, and 3. For the abovementioned reasons, we selected cases with clear bone exposure, represented as stages 1–3.

According to Çankaya *et al.* (13) the mean values of serum RANKL in our group with osteonecrosis were significantly lower than the corresponding control group (*p*=0.01). On the other hand, the mean values of serum OPG were higher in the osteonecrosis group than in the controls (*p*>0.01). These findings support the fact that BP treatment affects both osteoclasts and the RANKL/RANK/OPG signaling pathway. The RANKL/OPG ratio was lower in the group with osteonecrosis compared to the controls, suggesting that there were even more changes in OPG than in RANKL levels. Based on the abovementioned differences between the group with ONM and the controls, and considering the serum levels of RANKL and OPG, we explored whether the more advanced stages of osteonecrosis showed significant differences in the serum levels of RANKL and OPG compared to the early stages. Therefore, we explored whether serum levels of RANKL and OPG, and the RANKL/OPG ratio could be useful bio-markers for the severity of oral lesions. We did not observe significant differences among the three stages of osteonecrosis. Furthermore, serum levels of OPG did not show differences between patients with osteonecrosis treated intravenously for cancer compared to those treated orally for osteoporosis. Overall, serum levels of RANKL and OPG, and the RANKL/OPG RATIO were not valuable biomarkers for determining the severity of oral lesions in patients with osteonecrosis of the jaw.
